# The Mona Lisa Illusion—Scientists See Her Looking at Them Though She Isn’t

**DOI:** 10.1177/2041669518821702

**Published:** 2019-01-07

**Authors:** Gernot Horstmann, Sebastian Loth

**Affiliations:** Bielefeld University, Germany

**Keywords:** gaze direction, Mona Lisa effect, picture perception

## Abstract

If the person depicted in an image gazes at the camera or painter, a viewer perceives this as being gazed at. The viewers’ perception holds irrespectively of their position relative to image. This is the Mona Lisa effect named after the subject of Leonardo’s famous painting *La Gioconda*. The effect occurs reliably but was not tested with Mona Lisa herself. Remarkably, viewers judged Mona Lisa’s gaze as directed to their right-hand side irrespectively of the image zoom, its horizontal position on screen, and the distance of the ruler that was used for measuring the gaze direction.

Being photographed often triggers the subject to look straight into the camera. Thus, the typical family snapshot inevitably features someone staring at the viewer and stubbornly continuing to do so despite all attempts to move or rotate the photo or oneself. This effect was discovered in paintings and is known as the *Mona Lisa effect* named after the subject of the famous painting *La Gioconda* (Leonardo di ser Piero da Vinci, 1517). In brief, if the depicted person looks at the camera or painter, the viewer will feel being looked at irrespectively of their own position, distance, and angle relative to the image. Publications about the perception of gaze often assert that Mona Lisa gazes at the viewer but without presenting evidence (e.g., Al Moubayed, Edlund, & Beskow, 2012; Anstis, Mayhew, & Morley, 1969; Todorović, 2006). However, whether the participant feels (or has a strong desire of) being looked at is a judgement open to nonperceptual information including beliefs. The objective direction of gaze is not accessible to direct measurement, but its direct consequence, that is, the perceived line of gaze, can be measured. We asked for metric judgments, which are less prone to conflict with any beliefs than the binary judgement of beeing looked at. We conclude from the measurements that the lack of evidence is due to the claim being objectively false: Mona Lisa does not gaze at the viewer.

Parts of a high-resolution (7,479 px by 11,146 px) bitmap of the painting were presented on a 35 cm by 26 cm computer screen at a viewing distance of 66 cm. To test whether some aspects of Mona Lisa’s face may lead to different results, we manipulated whether only the eyes and nose or the entire head were visible to the participants. The image zoom ranged from 30% to 70% in steps of 10%. All images were centred to the same pixel on the bridge of the nose and cropped to match the screen resolution (1,280 px by 1,024 px), see centre column of Figure 2.

Instead of asking participants for the binary judgement of whether they feel being looked at, we obtained a metric of their perceived gaze direction on a 2 -m carpenter’s rule. The responses ranged from 0 to 200 avoiding negative numbers. The ruler was positioned between the participant and the painting (Anstis et al., 1969) and slightly off centre at 102.7 cm in order to avoid any bias to the even number (see Figure 1). We report the difference between a viewer-directed line of gaze and the participants’ judgement. Two points are required for computing the line of gaze and its angle. Thus, we switched the distance between ruler and screen from 15.5 cm to 35.5 cm or vice versa after half the trials (counterbalanced across participants).

**Figure 1.**
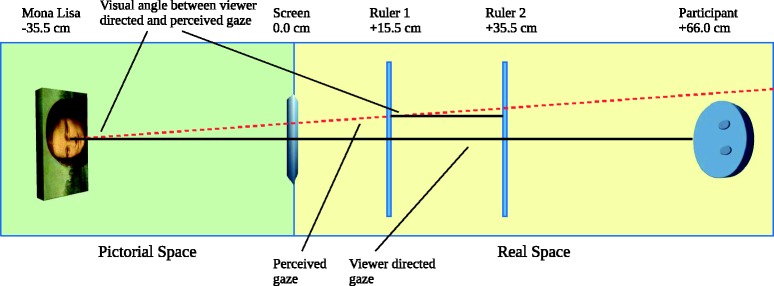
The red line connecting the intersections with Rulers 1 and 2 indicate Mona Lisa’s objective line of gaze (dashed). If Mona Lisa gazed at the viewer, the measurements and the perceived line of gaze would both intersect with the centre line (solid).

**Figure 2.**
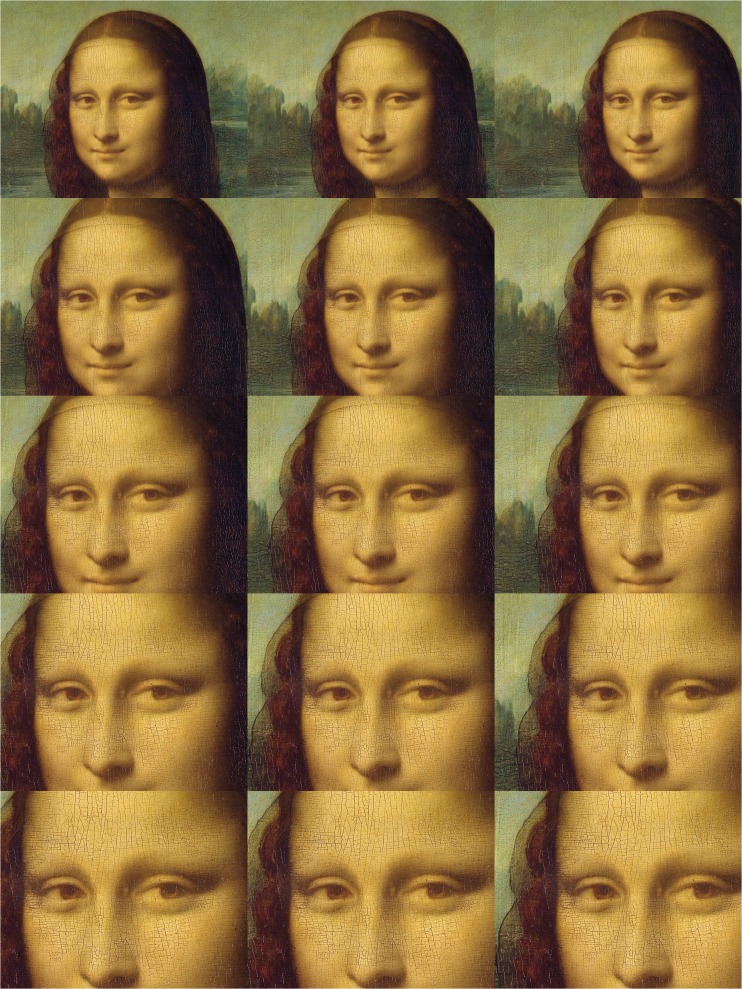
The widest (30%, top) and the narrowest (70%, bottom) zoom of the image sections used as stimuli with the respective lateral displacements.

This experiment required using a single stimulus rather frequently: *La Gioconda*. Thus, participants could be tempted to repeatedly enter the same number without assessing the stimulus. We addressed this by displacing the entire picture by 100 px (3.4 cm) to the left or right (Figure 2, left and right column). Twenty-four participants attended three repetitions of each combination of zoom and lateral displacement (randomised) for each screen–ruler distance.

If Mona Lisa gazed at the viewer, each point of measurement in Figure 3 would be located around zero for both ruler distances. If, however, Mona Lisa looked slightly sidewards, judgements should be different from zero and differ between both ruler distances (see Figure 1). Furthermore, the Mona Lisa effect predicts that she gazes at the viewer irrespective of lateral displacements of the image. Thus, the line of gaze would show a larger offset at Ruler 1 than at Ruler 2 (interaction of displacement and offset), whereas equal offsets are expected under the absence of the Mona Lisa effect (main effect of offset).

**Figure 3.**
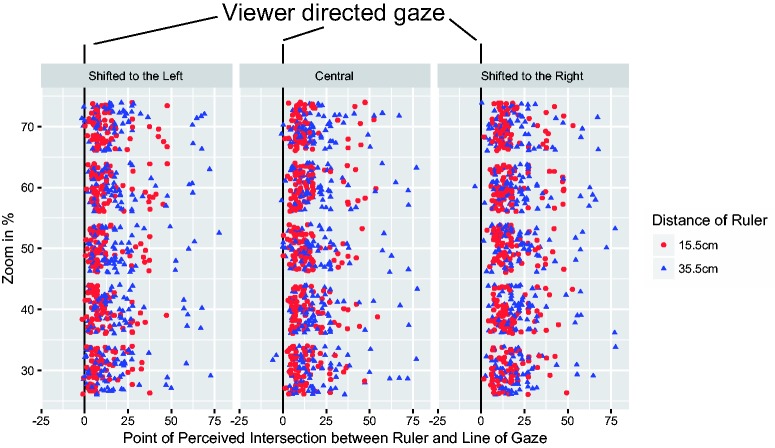
Each point marks the difference between the line of gaze directed at the participants and their responses in centimetre.

A Bayesian analysis of variance showed a main effect of distance (15.5 cm, 35.5 cm), ${\mathrm{BF}}_{10}=6\times 10^{5}$, and of displacement (left, centre, and right), ${\mathrm{BF}}_{10}=6\times 10^{5}$, and evidence against an interaction, ${\mathrm{BF}}_{10}=0.199$.

The significant increase in the judgements from 14.0 cm to 19.5 cm defines a line of gaze that is not directed at the viewer. These measures and the geometry in Figure 1 indicate that observers perceived a line of gaze with an angle of 15.4° to their right-hand side. This corresponds to 27.9 cm at their viewing distance of 66 cm. These results are the central tendency of a distribution of perceived gaze measurements reflecting uncertainty and error of measurement. Viewers can feel gazed at if their faces are inside this distribution that may cover up to 10° of visual angle in total or 5° to either side (Gamer & Hecht, 2007). However, Mona Lisa’s gaze angle of 15.4° is well outside that range and objectively contradicts the idea that Mona Lisa looks at the viewer.

The statistical evidence against an interaction of distance of the ruler and lateral displacement also contradicts the idea that Mona Lisa’s line of gaze was constantly directed at the viewer. Rather, the line of gaze and all its intersection points shifted with the image to the left or right, respectively.

A Bayesian linear regression revealed evidence against an effect of image zoom (30%, 40%, 50%, 60%, and 70%) on the participants’ judgements (${\mathrm{BF}}_{10}=0.161,R^{2}=.002$). Thus, the perception of depth was independent of the underlying image size (Rogers, 1995).

Our data suggest that Mona Lisa was consistently perceived as located 35.5 cm behind the screen in pictorial space at the intersection of a perpendicular line from her face and the perceived line of gaze (assuming no refraction at the boundary of pictorial real space).

Viewers move in real space and look into a fictional pictorial space through the window of a painting (cf. Rogers, 1995; Todorović, 2006). Manipulating the image zoom changes the size of this window but does not affect the perceived line of gaze. Moving the ruler forth and back extends or shortens the distance between the painting and the point of reference. This does not affect the line of gaze but enables us to determine its properties. We demonstrated that Mona Lisa gazes to her left-hand side from about 35.5 cm inside pictorial space and 15.4° to the viewer’s right-hand side in real space. Thus, Mona Lisa does not fulfil the premise of the Mona Lisa effect: She does not gaze at the viewer. Thus, the lateral shifts of the image resulted in a shift of the entire geometry rather than a constant gaze at the viewer as predicted by the effect. There is no doubt about the existence of the Mona Lisa effect—it just does not occur with Mona Lisa herself.

## References

[bibr1-2041669518821702] Al Moubayed S. Edlund J. Beskow J. (2012) Taming Mona Lisa: Communicating gaze faithfully in 2d and 3d facial projections. ACM Transactions on Interactive Intelligent Systems 1: 1–25. doi:10.1145/2070719.2070724.

[bibr2-2041669518821702] Anstis S. M. Mayhew J. W. Morley T. (1969) The perception of where a face or television ‘portrait’ is looking. The American Journal of Psychology 82: 474, doi:10.2307/1420441. 5398220

[bibr3-2041669518821702] Gamer M. Hecht H. (2007) Are you looking at me? Measuring the cone of gaze. Journal of Experimental Psychology: Human Perception and Performance 33: 705–715. doi:10.1037/0096-1523.33.3.705. 17563231 10.1037/0096-1523.33.3.705

[bibr4-2041669518821702] Leonardo di ser Piero da Vinci. (1517). *La Gioconda* [Oil on poplar panel]. Musée du Louvre, Paris, France.

[bibr5-2041669518821702] Rogers S. J. (1995) Perceiving pictoral space. In: Epstein W. Rogers S. J. (eds) *Perception of space and motion*, Handbook of perception and cognition, 2nd ed San Diego, CA: Academic Press, pp. 119–164.

[bibr6-2041669518821702] Todorović D. (2006) Geometrical basis of perception of gaze direction. Vision Research 46: 3549–3562. doi:10.1016/j.visres.2006.04.011. 16904157 10.1016/j.visres.2006.04.011

